# Jasmonate Zim-Domain Protein 9 Interacts With Slender Rice 1 to Mediate the Antagonistic Interaction Between Jasmonic and Gibberellic Acid Signals in Rice

**DOI:** 10.3389/fpls.2018.01866

**Published:** 2018-12-18

**Authors:** Tae Young Um, Han Yong Lee, Sangyool Lee, Sun Hyun Chang, Pil Joong Chung, Ki-Bong Oh, Ju-Kon Kim, Geupil Jang, Yang Do Choi

**Affiliations:** ^1^Department of Agricultural Biotechnology, Research Institute of Agriculture and Life Sciences, Seoul National University, Seoul, South Korea; ^2^Graduate School of International Agricultural Technology and Crop Biotechnology Institute, GreenBio Science and Technology, Seoul National University, Pyeongchang, South Korea; ^3^School of Biological Sciences and Technology, Chonnam National University, Gwangju, South Korea

**Keywords:** jasmonic acid, gibberellic acid, antagonistic interaction, OsJAZ9, SLR1, *Oryza sativa*

## Abstract

The jasmonic acid (JA) and gibberellic acid (GA) signaling pathways interact to coordinate stress responses and developmental processes. This coordination affects plant growth and yield, and is mediated by interactions between the repressors of each pathway, the JASMONATE ZIM-DOMAIN PROTEIN (JAZ) and DELLA proteins. In this study we attempted to identify rice (*Oryza sativa*) JAZs that interact with rice DELLAs such as SLENDER RICE 1 (SLR1). Analysis of protein–protein interactions showed that OsJAZ8 and OsJAZ9 interact with SLR1; OsJAZ9 also interacted with the SLR1-LIKE (SLRL) protein SLRL2. Based on this broader interaction, we explored the function of OsJAZ9 in JA and GA responses by analyzing transcript levels of the JA-responsive gene *OsbHLH148* and the GA-responsive gene *OsPIL14* in *OsJAZ9*-overexpressing (*OsJAZ9-Ox*) and *osjaz9* mutant plants. *OsbHLH148* and *OsPIL14* encode key transcription factors controlling JA and GA responses, respectively, and JA and GA antagonistically regulate their expression. In *OsJAZ9-Ox*, the expression of *OsbHLH148* was downregulated and the expression of *OsPIL14* was upregulated. By contrast, in *osjaz9* mutants, the expression of *OsbHLH148* was upregulated and the expression of *OsPIL14* was downregulated. These observations indicated that *OsJAZ9* regulates both JA and GA responses in rice, and this finding was supported by the opposite expression patterns of *OsDREB1s*, downstream targets of OsbHLH148 and OsPIL14, in the *OsJAZ9-Ox* and *osjaz9* plants. Together, these findings indicate that OsJAZ9 suppresses JA responses and promotes GA responses in rice, and the protein–protein interaction between OsJAZ9 and SLR1 is involved in the antagonistic interplay between JA and GA.

## Introduction

Plants coordinate their defenses and growth in response to environmental conditions; this process strongly affects growth and productivity in many crops, including rice, *Oryza sativa* (Hou et al., 2010; Yang et al., 2012; Jang et al., 2017). Previous studies showed that jasmonic acid (JA) or JA-dependent environmental stress responses affect the expression of genes involved in gibberellic acid (GA) responses (Navarro et al., 2008; Yang et al., 2012; Wild et al., 2012; Heinrich et al., 2013; Wild and Achard, 2013). These findings suggest that the interaction between JA and GA affects the coordination of defense responses and plant growth, which may have implications for developing crops with high-yield traits via manipulating the regulatory interactions between JA and GA.

JA is a key phytohormone that mediates plant responses to abiotic and biotic stresses and JA is synthesized from linolenic acid through the octadecanoid pathway (Creelman and Mullet, 1997; Stintzi, 2000; Wasternack and Hause, 2002; Farmer et al., 2003). In plant cells, JA is further metabolized into to a bioactive conjugated form, JA-isoleucine (JA-Ile), by the activity of JASMONATE RESISTANT 1 (JAR1) (Staswick and Tiryaki, 2004). In *Arabidopsis thaliana*, the interaction between JA-Ile and its receptor, CORONATINE INSENSITIVE1 (COI1), promotes JA signaling by provoking 26S proteasome-mediated degradation of the JASMONATE ZIM-DOMAIN (JAZ) repressor proteins (Xie et al., 1998; Thines et al., 2007; Fonseca et al., 2009). The proteolysis of JAZs leads to the release of transcription factors, such as MYC2 in Arabidopsis and OsbHLH148 in rice; these transcription factors induce the expression of JA-responsive genes. The *Arabidopsis thaliana* genome encodes 12 JAZ proteins (Lorenzo and Solano, 2005; Chini et al., 2007) and previous studies using overexpression and knock-down systems showed the essential role of *JAZs* in JA responses. For example, Shyu et al. (2012) showed that overexpression of *AtJAZ8* reduces the JA response (Shyu et al., 2012), and Yang et al. (2012) showed that overexpression of *JAZs* such as *AtJAZ1*, *AtJAZ3*, *AtJAZ9*, and *AtJAZ10* mimics the phenotype of *coi1* mutants, which have defects in JA signaling (Yang et al., 2012). By contrast, *AtJAZ1* or *AtJAZ10* knock-down plants were hypersensitive to JA (Yan et al., 2007; Grunewald et al., 2009). These observations indicate the important roles of JAZs in the JA response in Arabidopsis.

GA plays pivotal roles in many aspects of plant development such as plant height, leaf sheath growth, stem elongation, leaf expansion, flower development, and seed germination (Matsukura et al., 1998; Ikeda et al., 2001; Fu and Harberd, 2003; Cheng et al., 2004; Piskurewicz et al., 2008; Ubeda-Tomás et al., 2008; Achard et al., 2009; Qi et al., 2014). Similar to the JA signaling pathway, in the GA signaling pathway, proteolysis of the DELLA repressor proteins, is critical for the regulation of GA responses. In Arabidopsis, the interaction between GA and the GA INSENSITIVE DWARF1 (GID1) receptor provokes degradation of DELLAs through the 26S proteasome (Sasaki et al., 2003; Sun, 2011). This degradation leads to the release of transcription factors, such as PHYTOCHROME INTERACTING FACTORS (PIFs) 3 and 4 in Arabidopsis and PIF-LIKE (PIL) 13 and 14 in rice, which are responsible for inducing the expression of GA-responsive genes (Nakamura et al., 2007; De Lucas et al., 2008; Feng et al., 2008; Todaka et al., 2012; Cordeiro et al., 2016). The *Arabidopsis thaliana* genome contains 5 *DELLAs*, including the *REPRESSOR OF GA1-3* (*RGA*) gene. Plants that overexpress *RGA* show a reduced GA response and mutants lacking *RGA* exhibit enhanced GA responses, suggesting a crucial role of RGA in the GA response in Arabidopsis (Silverstone et al., 1998; Dill and Sun, 2001; King et al., 2001; Lee et al., 2002; Cheng et al., 2004).

The JA and GA signals interact synergistically or antagonistically in various aspects of plant development and defense. In Arabidopsis, JA and GA synergistically interact to regulate the development of stamen filaments and trichomes (Cheng et al., 2009; Qi et al., 2014), and a interaction between JAZs/DELLAs and the WD-repeat/bHLH/MYB complex is involved in the synergistic interaction between JA and GA in trichome development (Qi et al., 2014). JA interacts with GA antagonistically in hypocotyl elongation and root development (Hou et al., 2010; Yang et al., 2012; Hou et al., 2013). The interaction between JAZs and DELLAs attenuates their functions as signaling repressors. These reports indicate that the inhibitory interaction between JAZs and DELLAs plays a role in the coordination of defense and growth in Arabidopsis.

JA and GA signals also interact antagonistically in rice. For example, the suppression of plant height by JA is not observed in the *slender rice 1* (*slr1*) mutant, which lacks the activity of SLR1, a rice DELLA. This indicates that an antagonistic interaction between JA and GA strongly affects rice growth, and that *SLR1* is involved in this process (Yang et al., 2012). The expression pattern of *OsDREB1s*, which act downstream of the JA-responsive gene *OsbHLH148* and the GA-responsive gene *OsPIL14*, supports the antagonistic interaction between JA and GA, as *OsbHLH148* promotes *OsDREB1s* expression and *OsPIL14* suppresses *OsDREB1s* expression (Seo et al., 2011; Cordeiro et al., 2016). However, the molecular mechanism controlling the antagonistic interaction between JA and GA remains largely unknown in rice.

The *Oryza sativa* genome encodes 12 JAZs and 3 DELLAs, SLR1, SLRL1, and SLRL2 (Ikeda et al., 2001; Itoh et al., 2002; Tian et al., 2004; Ueguchi-Tanaka et al., 2007; Fukao and Bailey-Serres, 2008; Hirano et al., 2010). In this study, we found that OsJAZ8 and OsJAZ9 interact with SLRs, including SLR1. OsJAZ9 interacts with more SLRs than OsJAZ8, so we further investigated the role of OsJAZ9 in the regulation of JA and GA responses. Analysis of *OsJAZ9*-overexpressing and *osjaz9* mutant plants revealed that *OsJAZ9* differently regulates expression of *OsbHLH148* and *OsPIL14*, whose transcript levels are antagonistically regulated by JA and GA. These observations indicated that *OsJAZ9* is involved in antagonistic regulation of JA and GA responses in rice, and this finding was supported by the phenotype of *OsJAZ9*-overexpressing transgenic and *osjaz9* mutant plants. Taking these results together with the finding that OsJAZ9 interacts with SLR1, we propose that OsJAZ9 mediates the antagonistic interaction between stress-responsive JA signals and growth-promoting GA signals through interaction with SLR1 in rice.

## Materials and Methods

### Plant Materials, Growth Conditions, and Chemical Treatments

*Oryza sativa* L. cv. Dongjin was used as the wild-type rice. Rice seeds were germinated in one-half strength Murashige and Skoog (1/2 MS) agar medium in a growth chamber in the dark at 28°C for 3 days. After 3 days of germination, plants were grown in long-day conditions of 16-h light/8-h dark at 28°C for 14 days. For the GA and methyl jasmonate (MeJA) treatments, 8-week-old plants grown in soil were treated with 10 μM GA3 or 10 μM MeJA for the indicated times. The *osjaz9* knockout mutants were obtained by targeted CRISPR/Cas9 mutagenesis as previously described in Jang et al. (2016). The homozygous mutant *osjaz9-1*, which corresponds to line 2–3 and has a single nucleotide insertion at +194 bp from the start codon, and *osjaz9-2*, which corresponds to line 6–27 and has a single nucleotide insertion at +192 bp, were used in this study.

### Construction of Recombinant DNA Plasmids

For the construction of the *PGD1::OsJAZ9* and *PGD1::SLR1* plasmids, full-length *OsJAZ9* and *SLR1* cDNAs were amplified from total RNA by RT-PCR. The *OsJAZ9* and *SLR1* cDNAs were inserted into *BamH*I/*Sma*I and *Xba*I/*Sma*I-digested *pPZP200*, respectively; this vector contains the constitutively overexpressing *PHOSPHOGLUCONATE DEHYDROGENASE1* (*PGD1*) promoter (Park et al., 2010). The recombinant plasmid was verified by sequencing and then introduced into *Agrobacterium tumefaciens* LBA4404 for Agrobacterium-mediated rice transformation. Primer sequences are listed in Supplementary Table 1.

### Reverse Transcription-Quantitative Polymerase Chain Reaction (RT-qPCR)

RT-qPCR analyses were performed using total RNA extracted from the indicated plants. Extraction of total RNA was carried out using TRIzol (Invitrogen). For cDNA synthesis, 20 μL reactions were performed using 2 μg of total RNA, Superscript III reverse transcriptase, and oligo dT primers (Invitrogen). For quantitative PCR, a LightCycler 480 Instrument II Real-Time PCR machine with SYBR GREEN I Master Mix (Roche) was used. PCR conditions were programmed according to the manufacturer’s instructions (initial denaturation at 95°C for 5 min followed by 45 cycles of denaturation at 95°C for 10 s, annealing at 60°C for 10 s, and extension at 72°C for 10 s). *OsTubA2* (Os11g0247300) was used as an internal control. Three technical replicates of the RT-qPCRs were performed using three biological replicates. Primer sequences are listed in Supplementary Table 1.

### Yeast Two-Hybrid Assay

The Matchmaker Gold Yeast Two-Hybrid System (Clontech) was used to test the JAZ–DELLA interactions. Full-length cDNAs of *JAZ* and *DELLA* genes were amplified by RT-PCR from total RNA extracted from 14-day-old rice (Dongjin) or 2-week-old Arabidopsis (Col-0) plants. For construction of the bait plasmids, the *JAZs* were inserted into the Y2H bait vector *pGBKT7* (Clontech), which contains a tryptophan biosynthesis gene, *TRP1*, for selection. For construction of the prey plasmid, the *DELLA* genes were inserted into the Y2H prey vector *pGADT7* (Clontech), which contains a leucine biosynthesis gene, *LEU2*, for selection. The cDNAs of the *JAZs* and *DELLAs* were inserted using multiple enzyme sites (*Nde*I, *Eco*RI, and *BamH*I) of the *pGBKT7* and *PGADT7* vectors. The recombinant plasmids were co-transformed into the Y2H Gold yeast strain with the Aureobasidin A antibiotic resistance gene to test for protein–protein interactions. Co-transformed yeasts were selected in minimal yeast growth media without tryptophan and leucine (double dropout media, DDO). To test for the interactions between JAZs and DELLAs, co-transformed yeast (OD_600_ = 0.01) were placed on the DDO media including 250 ng/mL Aureobasidin A. After a 5-day incubation in the dark at 30°C, the growth of the yeast was captured using a Coolpix p300 (Nikon) digital camera. Sequences of the primers used for the bait and prey plasmids are provided in Supplementary Table 1.

### Protoplast Transformation and Co-immunoprecipitation (Co-IP) Assay

To analyze the interaction between OsJAZ9 and SLR1, OsJAZ9 and OsbHLH148 or SLR1 and OsPIL14, *pHBT-6xMyc*-*OsJAZ9*, *pHBT-GFP-SLR1*, *pHBT-GFP-OsbHLH148*, and *pHBT-6xMyc-OsPIL14* were generated by introducing amplified cDNAs of *OsJAZ9*, *SLR1*, *OsbHLH148*, and *OsPIL14* into *Stu*I/*Pst*I-digested *pHBT-5’6xMyc* or *Not*I/*Pst*I-digested *pHBT-5’GFP* plasmids using the Gibson assembly system (New England Biolabs). Rice protoplasts were isolated from 10-day-old rice seedlings (*O. sativa* cv. Donjin), and then co-transformed with *pHBT-6xMyc*-*OsJAZ9* and *pHBT-GFP-SLR1*, *pHBT-6xMyc*-*OsJAZ9* and *pHBT-GFP-OsbHLH148* or *pHBT-GFP-SLR1*, and *pHBT-6xMyc-OsPIL14* as previously described (Yang et al., 2014) with slight modification. Around 2.5 × 10^6^ protoplasts were co-transformed with 3 μg of *pHBT-6xMyc*-*OsJAZ9* and *pHBT-GFP-SLR1*, *pHBT-6xMyc*-*OsJAZ9* and *pHBT-GFP-OsbHLH148* or *pHBT-GFP-SLR1* and *pHBT-6xMyc-OsPIL14*. The co-transformed protoplasts were incubated in the dark at 28°C for 10 h, and then harvested by centrifugation at 300 g for 1 min. To extract total proteins, the protoplasts were homogenized with IP buffer 25 mM Tris-HCl (pH 7.5), 150 mM NaCl, 0.5 % Triton X-100, 1 mM EDTA, 1 mM DTT and Protease inhibitor cocktail (Roche) and then sonicated. For Co-IP, GFP antibody (Nacalai tesque) or Myc antibody (Santa Cruz Biotechnology) was added to the protein extracts and immunocomplexes were precipitated using 40 μl of Protein G Agarose Beads (Sigma). The immunocomplexes were isolated from the beads by boiling in sample buffer for 5 min and were separated using SDS-PAGE.

### Bimolecular Fluorescence Complementation (BiFC) Assay

The BiFC assay was used to test for the interaction between OsJAZ9 and SLR1 as described previously (Citovsky et al., 2006). Full-length cDNAs of *OsJAZ9* and *SLR1* were amplified by RT-PCR from total RNA extracted from 14-day-old rice (Dongjin). The amplified cDNA of *SLR1* was inserted into the *pSATN* vector and the cDNA of *OsJAZ9* was inserted into the *pSATC* vector using the *EcoR*I and *Sma*I sites (Tzfira et al., 2005). The *nYFP-SLR1* and *OsJAZ9*-*cYFP* constructs, empty *nYFP* and *OsJAZ9*-*cYFP* or *nYFP-SLR1* and empty *cYFP* were introduced into onion epidermal cells by tungsten particle bombardment as described by Sanford et al. (1987) with slight modifications. The *nYFP-SLR1* and *OsJAZ9*-*cYFP*, empty *nYFP* and *OsJAZ9*-*cYFP* or *nYFP-SLR1* and empty *cYFP* plasmids were mixed at a 1:1 (w/w) ratio and 50 μg of DNA was adsorbed onto 10 μg of 1-μm tungsten particles according to the manufacturer’s instructions (Bio-Rad). The particles were bombarded into onion epidermal cells at a pressure of 1000 kPa using a portable Helios gene gun system (model PDS-1000/He, Bio-Rad).

### Accession Numbers

Sequence data from this article can be found in the Rice Annotation Project (RAP) or the Arabidopsis Information Resource (TAIR) databases under the following accession numbers:*OsJAZ1* (Os10g0392400), *OsJAZ2* (Os03g0180900), *OsJAZ3* (Os03g0180800), *OsJAZ4* (Os03g0181100), *OsJAZ5* (Os03g0402800), *OsJAZ6* (Os07g0615200), *OsJAZ7* (Os09g0439200), *OsJAZ8* (Os09g0401300), *OsJAZ9* (Os08g0428400), *OsJAZ10* (Os04g0653000), *OsJAZ11* (Os04g0395800), *OsJAZ12* (Os02g0732400), *SLR1* (Os03g0707600), *SLRL1* (Os01g0646300), *SLRL2* (Os05g0574900), *OsPIL14* (Os07g0143200), *OsbHLH148* (Os03g0741100), *OsDREB1A* (Os09g0522200), *OsDREB1B* (Os09g0522000), *OsDREB1G* (Os02g0677300), and *RGA* (At2G01570).

## Results

### Identification of the Interactions Between OsJAZ8, OsJAZ9, and SLR1

**FIGURE 1:**
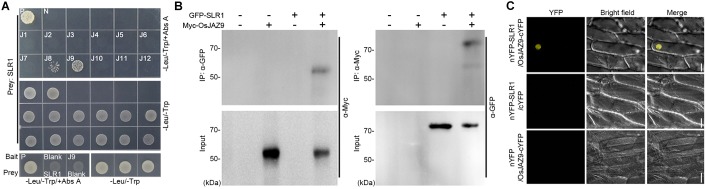
Analysis of the interaction between JAZ and DELLA proteins in rice. **(A)** A Y2H assay showing the interaction between OsJAZs and SLR1. Co-transformation with the p53 bait and T prey plasmid was used for a positive control (P) and co-transformation with the LAM bait and T prey plasmid was used for a negative control (N). -Leu/-Trp/+Abs A indicates aureobasidin A-containing DDO media to test for the JAZs-DELLA interaction. -Leu/-Trp means DDO media to verify yeast transformation of the indicated bait and prey plasmid. J1–J12 indicates the OsJAZ1–OsJAZ12 bait plasmids used for yeast transformation. Blank indicates empty bait or prey plasmid. **(B)** Co-IP results showing the interaction between OsJAZ9 and SLR1. Total proteins were extracted from rice protoplasts co-transformed with *6xMyc-OsJAZ9* and *GFP-OsSLR1*. IP indicates immunoprecipitation, and α-GFP (left) and α-Myc (right) indicate the antibodies used for the immunoprecipitation. α-Myc (left) and α-GFP (right) are the antibodies used for western blotting to detect the interaction between OsJAZ9 and SLR1. **(C)** A bimolecular fluorescence complementation (BiFC) assay showing the interaction between OsJAZ9 and SLR1 protein. Onion epidermal cells were co-transfected with *nYFP-SLR1* and *OsJAZ9-cYFP* (top), *nYFP-SLR1* and empty *cYFP* (middle), or empty *nYFP* and *OsJAZ9-cYFP* (bottom) plasmids by tungsten particle bombardment. The yellow channel image is shown in the YFP panel and the bright-field panel shows the differential interference contrast image. Scale bars = 50 μm.

To explore the interaction between JAZs and DELLA proteins in rice, we performed yeast two-hybrid (Y2H) assays using the 12 OsJAZs as bait and SLR1 as prey (Figure 1A). The yeast cells (*Saccharomyces cerevisiae* Y2H GOLD) co-transformed with the *OsJAZ8* or *OsJAZ9* bait plasmid together with the *SLR1* prey plasmid survived in media containing aureobasidin A (Abs A), which selects for yeast lines with a direct interaction between the bait and prey proteins (Chang et al., 2017). Yeast lines transformed with other *OsJAZ* bait plasmids did not survive on Abs A. These results indicated that OsJAZ8 and OsJAZ9 interact with rice SLR1. In Arabidopsis, six JAZs, AtJAZ1, 3, 4, 9, 10, and 11, interact with the Arabidopsis DELLA protein RGA (Hou et al., 2010; Yang et al., 2012). Examination of the interaction between RGA and the 12 OsJAZs showed that RGA interacted only with OsJAZ8 and OsJAZ9, as did SLR1 (Supplementary Figure 1).

To verify the interaction between OsJAZ9 and SLR1 *in vivo*, we performed Co-IP assays using rice protoplasts co-transformed with *6xMyc-OsJAZ9* and *GFP-SLR1* (Figure 1B). When protein extracts were immunoprecipitated with anti-GFP antibody, 6xMyc-OsJAZ9 was co-immunoprecipated with GFP-SLR1. Similar results were obtained by immunoprecipitating 6xMyc-OsJAZ9 with anti-Myc antibody, indicating that OsJAZ9 interacts with SLR1. Bimolecular fluorescence complementation (BiFC) assays using *nYFP-SLR1* and *OsJAZ9-cYFP* plasmids further supported the interaction between OsJAZ9 and SLR1 (Figure 1C). When these plasmids were introduced into onion epidermal cells by tungsten particle bombardment, fluorescent signals were observed in the nucleus. However, the onion epidermal cells co-transformed with *nYFP-SLR1* and empty *cYFP*, or empty *nYFP* and *OsJAZ9-cYFP* did not show YFP signals. These observations showed that the JA signaling repressor OsJAZ9 and the GA signaling repressor SLR1 interact.

We also tested the interaction between OsJAZs and other rice DELLA proteins such as SLRL1 and SLRL2. SLRL1 did not interact with any of the OsJAZs in the Y2H assay and SLRL2 interacted with OsJAZ9 but not with the other OsJAZs (Supplementary Figure 2). This showed that OsJAZ9 has a broader interaction with SLR proteins compared with OsJAZ8 proteins.

### The N-Terminal Region of OsJAZ9 Interacts With the C-Terminal Region of SLR1

**FIGURE 2:**
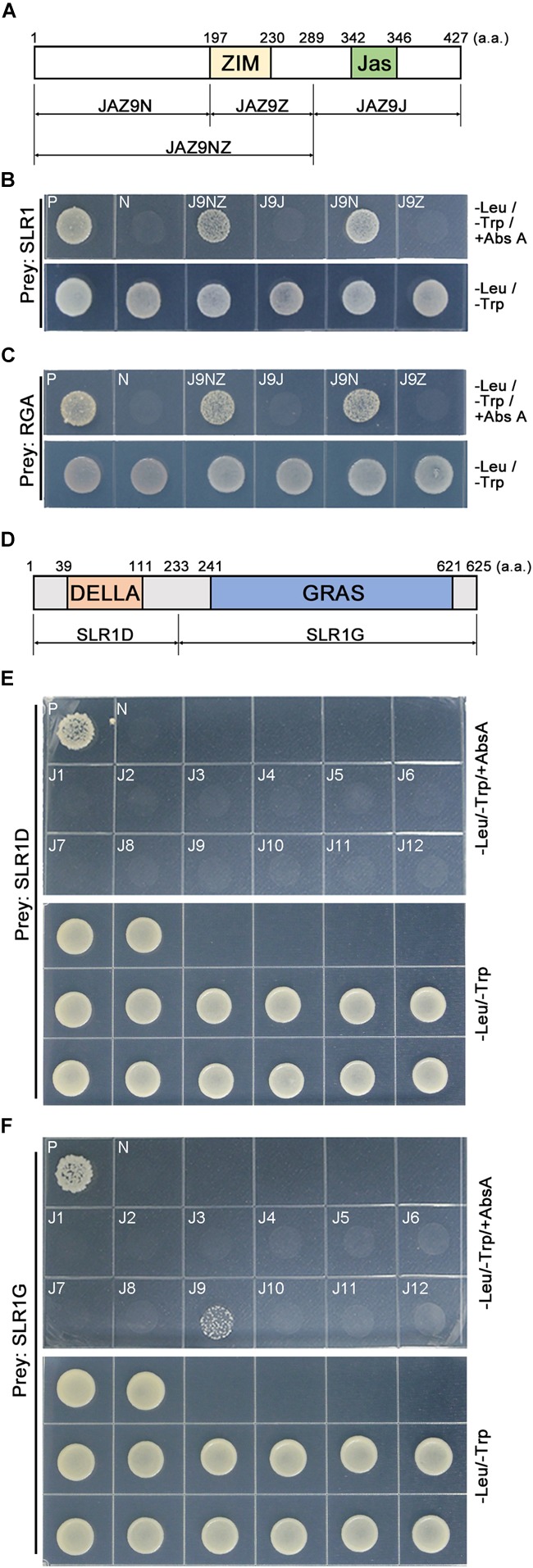
Identification of the domains in OsJAZ9 and SLR1 proteins that mediate their interaction. **(A)** Schematic of the truncated OsJAZ9 proteins used to analyze the interaction with DELLA proteins. The colored boxes indicate the ZIM (yellow) and Jas (green) domains (Seo et al., 2011). **(B,C)** Truncated *OsJAZ9* bait constructs were co-transformed with *SLR1* **(B)** or Arabidopsis *RGA* (At2G01570) **(C)** prey constructs and plated out on DDO media containing Abs A. -Leu/-Trp/+Abs A and -Leu/-Trp indicate DDO media with and without aureobasidin A, respectively. **(D)** Schematic of the truncated SLR1 proteins used to test the interaction with JAZ proteins. The colored boxes indicate the DELLA (orange) and GRAS (blue) domains (Ueguchi-Tanaka et al., 2007; Hirano et al., 2010). **(E,F)** Yeast lines were co-transformed with *OsJAZ* bait plasmids (J1–J12) together with truncated *SLR1D* **(E)** or *SLR1G* **(F)** prey plasmids and plated on Abs-A containing media.

To understand how OsJAZ9 interacts with SLR1, we generated a series of *OsJAZ9* bait plasmids encoding truncated OsJAZ9 proteins and carried out Y2H assays (Figures 2A,B). The yeast line co-transformed with the *SLR1* prey plasmid and the truncated *JAZ9N* bait, which encodes the N-terminal region upstream of the ZIM domain, survived on the Abs A media. The yeast line co-transformed with the *SLR1* prey plasmid and the *JAZ9NZ* bait plasmid, which encodes the N-terminal region and the ZIM domain, also survived on the Abs A media. However, the yeast lines carrying *JAZ9Z* and *JAZ9J*, without the N-terminal region, as baits did not survive in this condition. The N-terminal region of OsJAZ9 also interacted with RGA proteins (Figure 2C). These observations indicated that the N-terminal region of OsJAZ9 is responsible for its interaction with DELLA proteins.

To identify the SLR1 domain responsible for its interaction with OsJAZ9, we generated two types of truncated *SLR1* prey encoding SLR1D and SLR1G, which contain the DELLA domain and the GRAS domain, respectively (Figure 2D). In the Y2H assay, we found that the yeast transformed with the truncated *SLR1G* prey plasmid together with the *OsJAZ9* bait plasmid survived on the Abs A media. However, the yeast line carrying the *SLR1D* prey plasmid did not survive on Abs A (Figures 2E,F). These results showed that the interaction between OsJAZ9 and SLR1 is mediated through the N-terminal region of OsJAZ9 and the GRAS domain of SLR1 in rice.

### OsJAZ9 and SLR1 Show Similar Expression Patterns

**FIGURE 3:**
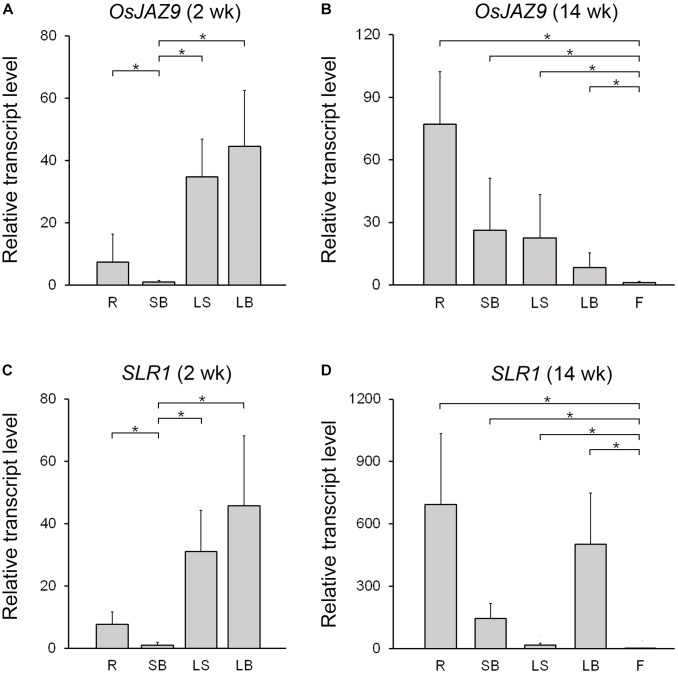
Expression patterns of *OsJAZ9* and *SLR1* in rice. **(A,B)** Expression of *OsJAZ9* in 2- **(A)** and 14-week-old rice **(B)**. **(C,D)** Expression patterns of *SLR1* in 2- **(C)** and 14-week-old rice **(D)**. Tissues tested were: root (R), shoot base (SB), leaf sheath (LS), leaf blade (LB), and flower (F). Total RNAs were extracted from the indicated samples, and the expression level was analyzed by RT-qPCR. Data represent mean values of three biological replicates, and error bars indicate SD. Asterisks show statistically significant differences between the indicated samples (*p*-value < 0.01, Student’s *t*-test). *OsTubA2* (Os11g0247300) was used as an internal control and relative expression levels are shown as fold values.

To investigate the spatial expression patterns of *OsJAZ9* and *SLR1* in rice, we extracted total RNAs from the root, shoot base, leaf sheath, leaf blade, and flower and measured the transcript levels of these genes by RT-qPCR (Figure 3). In 2-week-old plants, the expression of *OsJAZ9* was higher in the leaf sheath and leaf blade than in the other tissues (Figure 3A). However, in 14-week-old plants, the roots exhibited higher expression of *OsJAZ9* than the leaves and flowers (Figure 3B), indicating that tissue-specific expression of *OsJAZ9* changes as the plant develops.

If the interaction between OsJAZ9 and SLR1 is involved in crosstalk between JA and GA and the coordination of plant defense and growth, it would be expected that *SLR1* and *OsJAZ9* would have similar patterns of expression during development. To test this idea, we analyzed the expression pattern of *SLR1* in the root, shoot base, leaf sheath, leaf blade, and flower of 2- and 14-week-old plants. The expression of *SLR1* was higher in the leaf sheath and leaf blade than in the other tissues in 2-week-old plants (Figure 3C). In 14-week-old plants, the level of *SLR1* mRNA was higher in the roots than in the leaves, as was *OsJAZ9* (Figure 3D). These results show that the spatial and temporal expression patterns of *OsJAZ9* and *SLR1* are similar during rice development, consistent with the hypothesis that these two genes may function in the same tissues.

### Expression of OsbHLH148, OsPIL14, and OsDREB1s Is Antagonistically Regulated by JA and GA

Previous studies in Arabidopsis showed that the interaction between JAZs and DELLAs regulates the antagonistic interplay between JA and GA (Hou et al., 2010; Yang et al., 2012). To understand the function of *OsJAZ9* in JA and GA responses, we attempted to identify genes that are antagonistically regulated by JA and GA. Similar to the previous results with Arabidopsis *JAZs* and *RGA*, we found that the transcript levels of *OsJAZ9* and *SLR1* were not antagonistically regulated by JA and GA (Supplementary Figure 3).

**FIGURE 4:**
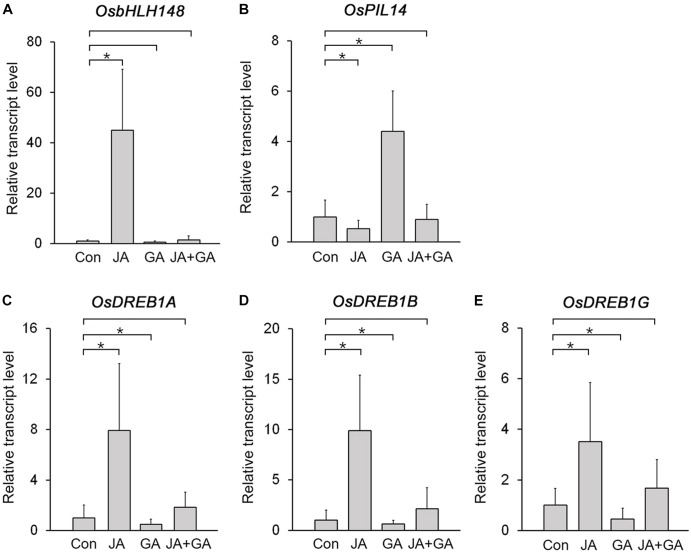
Identification of rice genes antagonistically regulated by JA and GA. **(A,B)** RT-qPCR analysis showing changes in transcript levels of *OsbHLH148* **(A)** and *OsPIL14* **(B)** in the leaf blade of 8-week-old WT plants (Dongjin) treated with 10 μM MeJA (JA), 10 μM GA3 (GA), or 10 μM MeJA + 10 μM GA3 (JA+GA) for 3 h. **(C–E)** RT-qPCR analysis showing changes in transcript levels of *OsDREB1A* **(C)**, *OsDREB1G* **(D)**, and *OsDERB1G* **(E)** in the leaf blades of 8-week-old WT plants (Dongjin) treated with 10 μM MeJA (JA), 10 μM GA3 (GA), or 10 μM MeJA + 10 μM GA3 (JA+GA) for 3 h. Control plants (Con) were not treated with JA, GA, or JA+GA. Data represent mean values of three biological replicates, and error bars indicate SD. Asterisks indicate statistically significant differences between the corresponding samples and their control (*p*-value < 0.01, Student’s *t*-test). *OsTubA2* was used as an internal control and relative expression levels are shown as fold values.

The JA-responsive gene *OsbHLH148* and the GA-responsive gene *OsPIL14* encode key transcription factors controlling JA and GA responses in rice, and their expression is deeply involved in plant growth and stress responses (Seo et al., 2011; Du et al., 2013; Cordeiro et al., 2016). Because OsbHLH148 promotes the expression of drought-induced genes, such as *OsDREB1A*, *OsDREB1B*, and *OsDREB1G*, but OsPIL14 represses the expression of *OsDREB1B* (Seo et al., 2011; Cordeiro et al., 2016), we expected that expression of *OsbHLH148* and *OsPIL14* would be antagonistically regulated by JA and GA. To test this, we analyzed changes in their transcript levels in response to JA and GA treatments (Figures 4A,B; Supplementary Figure 4). The transcript level of *OsbHLH148* was strongly increased by the JA treatment, but slightly reduced by the GA treatment. The transcript level of *OsHLH148* in the plants co-treated with JA and GA was much lower than that in the plants treated with JA alone. This indicated that GA nullifies the effect of JA on *OsbHLH148* expression (Figure 4A). For *OsPIL14*, the GA treatment increased *OsPIL14* expression and the JA treatment reduced *OsPIL14* expression. However, JA+GA co-treatment negated the effects of both JA and GA on the expression of *OsPIL14* (Figure 4B). These results showed that the expression of *OsbHLH148* and *OsPIL14* is antagonistically regulated by JA and GA.

If JA and GA antagonistically regulate the expression of *OsbHLH148* and *OsPIL14*, then expression of *OsDREB1s* should also be antagonistically regulated by JA and GA, because OsbHLH148 activates *OsDREB1s* and OsPIL14 suppresses *OsDREB1s* (Seo et al., 2011; Cordeiro et al., 2016). As expected, the expression of *OsDREB1s* was upregulated in response to JA, but downregulated by GA. The JA+GA co-treatment diminished the effects of JA and GA on the expression of *OsDREB1s*, as was observed with *OsbHLH148* and *OsPIL14* (Figures 4C–E), supporting the idea that expression of *OsbHLH148* and *OsPIL14* was antagonistically regulated by JA and GA. In addition, analysis of protein–protein interactions using Co-IP showed that OsJAZ9 and SLR1 interact with OsbHLH148 and OsPIL14, respectively (Supplementary Figure 5). This finding indicates that OsbHLH148 and OsPIL14 are involved in the OsJAZ9 and SLR1-mediated antagonistic interplay between JA and GA in rice.

### Overexpression of *OsJAZ9* Promotes the GA Response

**FIGURE 5:**
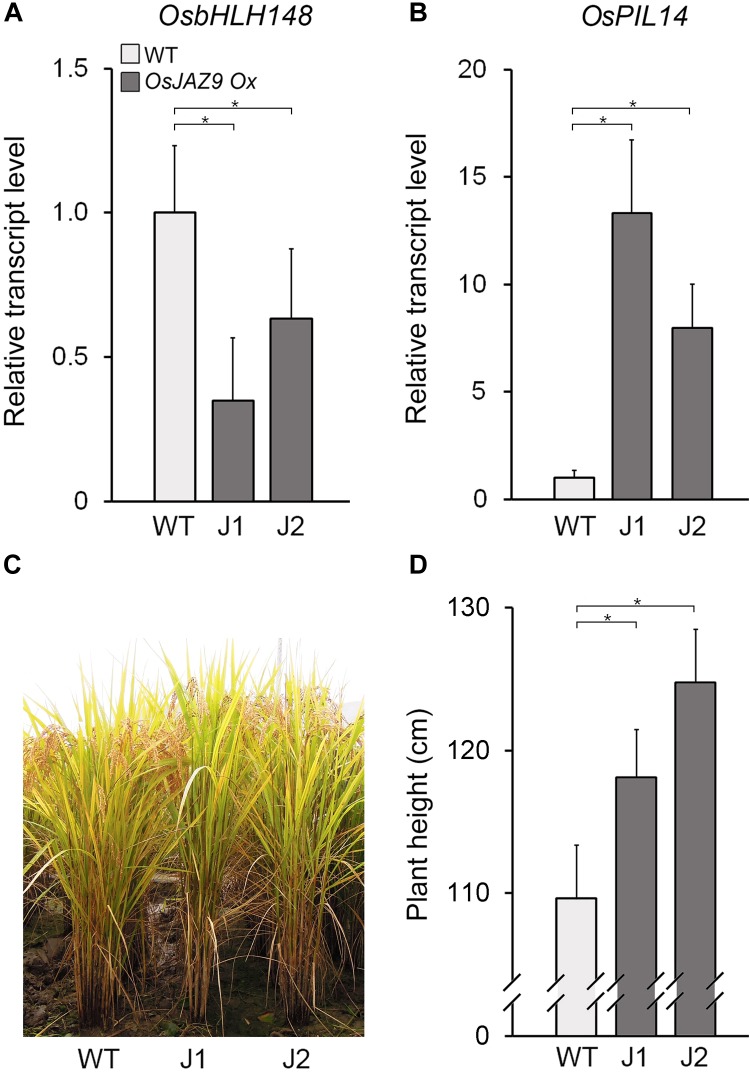
Overexpression of *OsJAZ9* promotes the GA response in rice. **(A,B)** RT-qPCR analysis showing the expression levels of the JA-responsive gene *OsbHLH148* **(A)** and the GA-responsive gene *OsPIL14* **(B)** in the leaf blades of 8-week-old *OsJAZ9*-overexpressing transgenic rice (*PGD1::OsJAZ9*). J1 and J2 indicate two independent lines of *PGD1::OsJAZ9* plants. Data represent mean values of three biological replicates, and error bars indicate SD. *OsTubA2* was used as an internal control and relative expression levels are shown as fold values. **(C,D)** Growth of *PGD1::OsJAZ9* plants grown in field conditions for 25 weeks (C), and quantification of plant height **(D)** (*n* > 30). Error bars indicate SD. Asterisks indicate statistically significant differences between the corresponding samples and their control (*p*-value < 0.01, Student’s *t*-test).

To understand the function of *OsJAZ9* in the antagonistic interaction between JA and GA, we analyzed the effect of *OsJAZ9* overexpression on the expression of *OsbHLH148* and *OsPIL14*. To do this, we generated transgenic rice lines overexpressing *OsJAZ9* under the control of the constitutively overexpressing *PHOSPHOGLUCONATE DEHYDROGENASE1* (*PGD1*) promoter (Supplementary Figure 6; Park et al., 2010). We identified two independent lines (lines 10 and 15) of transgenic plants with a single-copy insertion of the *PGD1::OsJAZ9* transgene (Supplementary Figure 6A). Expression levels of *OsJAZ9* in these transgenic plants were approximately 6–11 fold higher than that in wild-type plants (Supplementary Figure 6B). In these *OsJAZ9-*overexpressing transgenic plants (*OsJAZ9-Ox*), the transcript levels of *OsbHLH148* were downregulated (Figure 5A), indicating that overexpression of *OsJAZ9* reduces the JA response in rice.

These findings were partially supported by the phenotype of the *OsJAZ9-Ox* plants, which had longer roots compared with wild-type plants (Supplementary Figure 7). Furthermore, the length of the *OsJAZ9-Ox* plants relative to wild type increased in response to JA. This indicated that *OsJAZ9-Ox* plants are less sensitive to JA than wild-type plants. In contrast to the expression of JA-responsive *OsbHLH148*, the expression of the GA-responsive gene *OsPIL14* was higher in the *OsJAZ9-Ox* plants than in wild-type plants (Figures 5B), and the *OsJAZ9-Ox* plants were taller than wild type (Figures 5C,D). Because the GA response promotes rice growth (Huang et al., 1998; Matsukura et al., 1998), these results indicate that the taller phenotype in the *OsJAZ9-*overexpressing transgenic plants resulted from an enhanced GA response.

### The GA Response Is Reduced in the *osjaz9* Knockout Mutants

**FIGURE 6:**
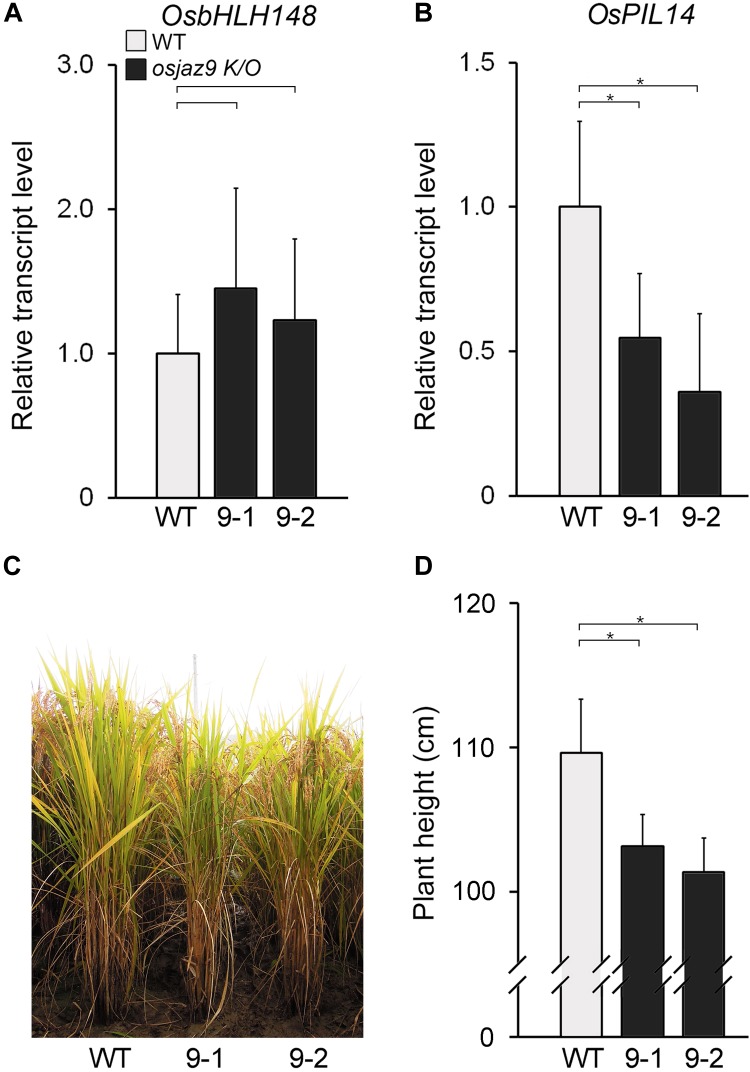
The reduced GA response in the *osjaz9* mutants. **(A,B)** RT-qPCR analysis showing the expression levels of *OsbHLH148* **(A)** and *OsPIL14* **(B)** in the leaf blades of 8-week-old *osjaz9* mutant plants. 9-1 and 9-2 indicate two independent mutants, *osjaz9-1*, and *osjaz9-2*. Data represent mean values of three biological replicates, and error bars indicate SD. *OsTubA2* was used as an internal control and relative expression levels are shown as fold values. **(C,D)** Images of *osjaz9* plants grown in field conditions for 25 weeks **(C)**, and quantification of plant height **(D)** (*n* > 30). Error bars indicate SD. Asterisks indicate statistically significant differences between the corresponding samples and their control (*p*-value < 0.01, Student’s *t*-test).

If *OsJAZ9* regulates both JA and GA responses, the mutant rice line lacking OsJAZ9 activity would be expected to exhibit some changes in the JA and GA responses. To address this, we analyzed the JA and GA responses in two independent *osjaz9* knockout mutants (*osjaz9-1* and *osjaz9-2*), generated by CRISPR/Cas9 (Jang et al., 2016). In both of the independent *osjaz9* knockout mutants, the expression of the JA-responsive gene *OsbHLH148* tended to be higher than that in the wild-type plants, but not significantly so (Figure 6A). The root growth of *osjaz9* mutants was similar to that of wild-type plants in JA-untreated conditions (Supplementary Figure 8). However, in JA-treated conditions, the root length of *osjaz9* knockout mutants was significantly shorter than that of wild-type plants, indicating that *osjaz9* mutants are more sensitive to JA than wild-type plants. In contrast to the expression of *OsbHLH148*, the expression of the GA-responsive gene *OsPIL14* was lower in the *osjaz9* knockout mutants than in the wild-type plants (Figures 6B). Furthermore, the *osjaz9* knockout mutants were shorter than the wild-type plants (Figures 6C,D). These findings indicate that the *osjaz9* plants exhibit reduced GA responses compared to the wild-type plants. Together with the results of the *OsJAZ9*-overexpressing plants, these observations supported the idea that OsJAZ9 antagonistically regulates the JA and GA responses.

To verify this, we further analyzed the expression of *SLR1* and *OsDREB1s* in *OsJAZ9*-*Ox* and *osjaz9* knockout mutant plants (Supplementary Figure 9). Unlike *SLR1*, whose expression was not antagonistically regulated by JA and GA, the *OsDREB1s* showed opposite expression patterns in the *OsJAZ9*-*Ox* plants and the *osjaz9* mutants: the *OsJAZ9*-*Ox* plants exhibited reduced expression of *OsDREB1s* and the *osjaz9* mutants showed increased expression of *OsDREB1s.* These findings supported the hypothesis that *OsJAZ9* modulates the antagonistic interplay between JA and GA signaling in rice, and the interaction between OsJAZ9 and OsSLR1 is deeply involved in this process.

## Discussion

In this study, we found that OsJAZ8 and OsJAZ9, 2 of the 12 OsJAZs, interact with SLRs. By contrast, 6 of the 12 Arabidopsis JAZs interact with RGA (Hou et al., 2010; Yang et al., 2012). Therefore, compared with Arabidopsis, the interaction between OsJAZs and SLRs is simpler and more specific, because fewer OsJAZs interact with each SLR. The ability of OsJAZ8 and OsJAZ9 to interact with DELLAs was supported by phylogenetic analysis (Supplementary Figure 10). OsJAZ8 and OsJAZ9 are the most similar to each other of the OsJAZs, and are closely related to AtJAZ1, 3, 4, 9, 10, and 11, which interact with RGA. Although OsJAZ8 and OsJAZ9 interact with rice SLR1 and Arabidopsis RGA, only OsJAZ9 interacted with SLRL2. The broader interaction of OsJAZ9 proteins with SLRs indicates that OsJAZ9 might be largely responsible for the interplay between JA and GA through the interaction with DELLA proteins in rice, and the Co-IP and BiFC results showing the interaction between OsJAZ9 and SLR1 protein support this idea.

In this study, we examined the transcript levels of the JA-responsive gene *OsbHLH148* and the GA-responsive gene *OsPIL14* in *OsJAZ9-Ox* plants and *osjaz9* knockout mutants to understand the function of *OsJAZ9* in the regulation of JA and GA responses. In rice, OsbHLH148 and OsPIL14 play essential roles in the regulation of JA and GA responses (Seo et al., 2011; Cordeiro et al., 2016). We found that the transcript levels of *OsbHLH148* and *OsPIL14* are antagonistically regulated by JA and GA. This indicated that regulation of the expression of *OsbHLH148* and *OsPIL14* is involved in the antagonistic interplay between JA and GA in rice, and the finding that OsbHLH148 and OsPIL14 interact with OsJAZ9 and SLR1, respectively, supported this. When the transcript levels of *OsbHLH148* and *OsPIL14* were measured in *OsJAZ9-Ox* and *osjaz9* knockout mutants, we found that *OsJAZ9-Ox* plants exhibited decreased expression of *OsbHLH148*, but increased expression of *OsPIL14* while *osjaz9* plants showed increased expression of *OsbHLH148*, but decreased expression of *OsPIL14*. These findings indicate that *OsJAZ9* antagonistically regulates JA and GA responses. These findings were supported by characterization of *SLR1*-overexpressing plants (*SLR1-Ox*) (Supplementary Figure 11). Similar to overexpression of *OsJAZ9*, overexpression of *SLR1* affected the expression of *OsbHLH148* and *OsPIL14*. However, the expression pattern was opposite between *OsJAZ9-Ox* and *SLR1-Ox* plants: in *SLR1*-overexpressing plants, the expression of *OsbHLH148* was upregulated while the expression of *OsPIL14* was downregulated. Taking these results together with the finding that OsJAZ9 interacts with SLR1, we suggest that OsJAZ9 antagonistically regulates the JA and GA responses in rice, and the interaction between OsJAZ9 and SLR1 is deeply involved in this process.

## Author Contributions

YC and GJ conceived the original screening and research plans. TU performed most of the experiments. HL, SL, SC, and PC provided technical assistance to TU. YC, K-BO, and GJ analyzed the data. GJ, J-KK, and YC wrote the article with contributions of all the authors.

## Conflict of Interest Statement

The authors declare that the research was conducted in the absence of any commercial or financial relationships that could be construed as a potential conflict of interest.
