# Supplementary material for: Jasmonate Zim-Domain Protein 9 Interacts With Slender Rice 1 to Mediate the Antagonistic Interaction Between Jasmonic and Gibberellic Acid Signals in Rice
Source: Front Plant Sci. 2018 Dec 18;9:1866. doi: 10.3389/fpls.2018.01866 (PMC6305323; doi:10.3389/fpls.2018.01866)
Supplement: Supplementary file 1 [file Data_Sheet_1.PDF]

*Supplementary Material*

# JASMONATE ZIM-DOMAIN PROTEIN 9 interacts with SLENDER RICE 1 to mediate the antagonistic interaction between jasmonic and gibberellic acid signals in rice

Tae Young Um, Han Yong Lee, Sangyool Lee, Sun Hyun Chang, Pil Joong Chung, Ki-Bong Oh, Ju-Kon Kim, Geupil Jang*, and Yang Do Choi*

*** Correspondence:** Yang Do Choi: choiyngd@snu.ac.kr and Geupil Jang: geupil.jang@gmail.com

**Supplementary Figures**

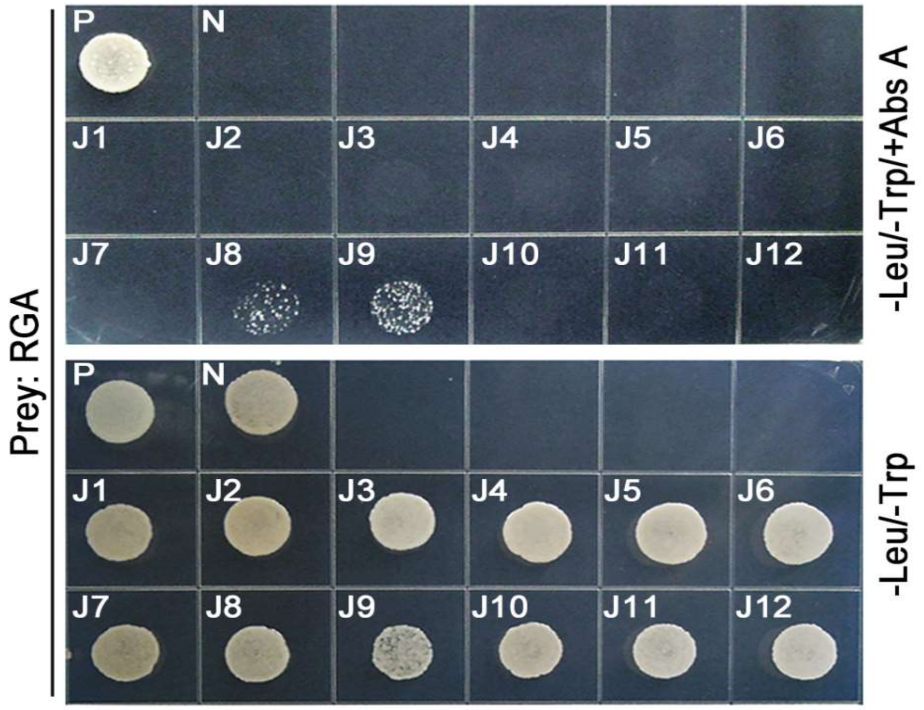


**Supplementary Figure 1.** Interaction between OsJAZs and RGA.
Y2H assay showing the interaction between an Arabidopsis DELLA protein (RGA) and OsJAZs. Co-transformation with the p53 bait and T prey plasmids was used for a positive control (P) and co-transformation with the LAM bait and T prey plasmids was used for a negative control (N). -Leu/-Trp/+Abs A indicates aureobasidin A-containing double dropout media (DDO) to test for JAZ–DELLA interactions (top). -Leu/-Trp is the DDO without leucine and tryptophan used for verification of yeast transformation and equal dropping (bottom). J1–J12 indicates OsJAZ1–OsJAZ12 bait plasmids.

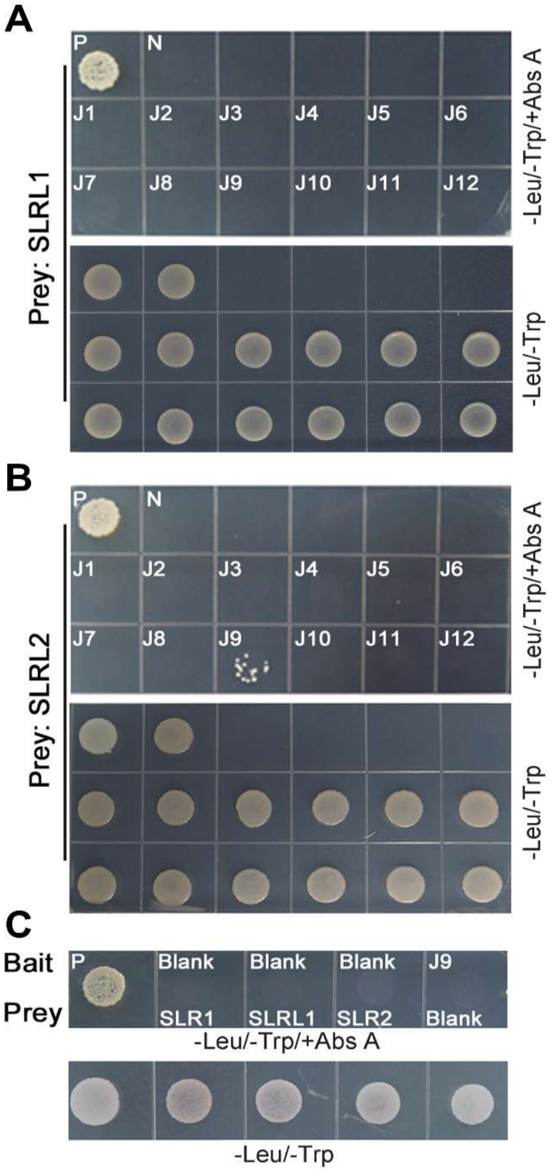


**Supplementary Figure 2.** Interaction between OsJAZs and SLRL1 or SLRL2.
**(A, B)** Y2H assay showing the interaction between OsJAZs and SLRL1 **(A)** or SLRL2 **(B)**. Co-transformation with the p53 bait and T prey plasmid was used for a positive control (P) and co-transformation with the LAM bait and T prey plasmid was used for a negative control (N). **(C)** Y2H control results supporting the OsJAZ9–SLRL2 interaction. -Leu/-Trp/+Abs A indicates aureobasidin A-containing DDO media used to test for the JAZ–DELLA interaction. -Leu/-Trp indicates DDO media used to verify yeast transformation of the indicated bait and prey plasmid. J1–J12 indicates the OsJAZ1–OsJAZ12 bait plasmids used for yeast transformation. Blank indicates empty bait or prey plasmid.

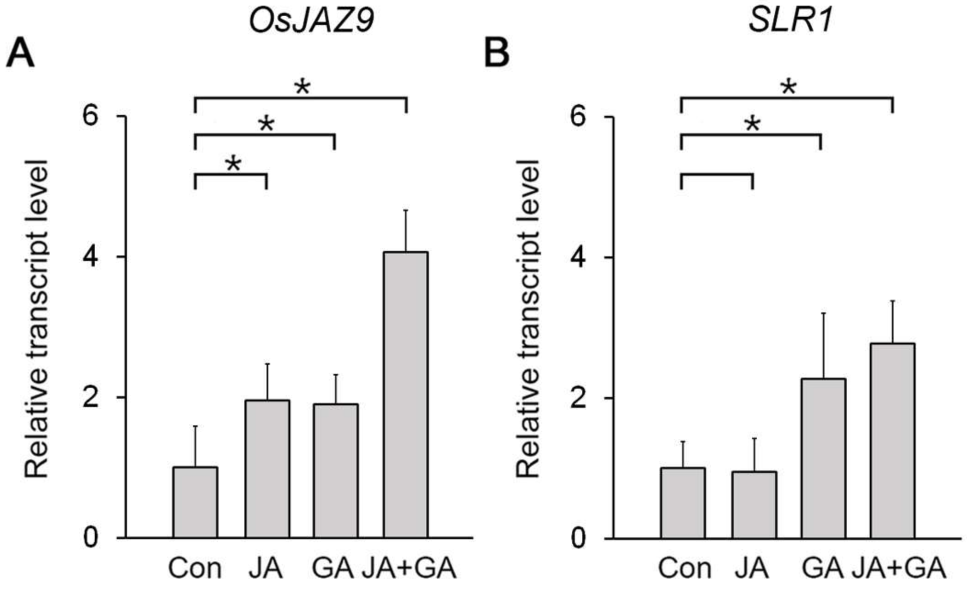


**Supplementary Figure 3.** Expression of *OsJAZ9* and *SLR1* in response to JA and GA.
**(A, B)** RT-qPCR analysis showing changes in transcript levels of *OsJAZ9* **(A)** and *SLR1* **(B)** in the leaf blade of 8-week-old WT plants (Dongjin) treated with 10 µM MeJA (JA), 10 µM GA3 (GA), or 10 µM MeJA + 10 µM GA3 (JA+GA) for 3 hrs. Control plants (Con) were not treated with JA, GA, or JA+GA. Data represent mean values of three biological replicates, and error bars indicate SD. Asterisks indicate statistically significant differences between the corresponding samples and their control ($p$ value < 0.01, Student's $t$-test). *OsTubA2* was used as an internal control and relative expression levels are shown in fold values.

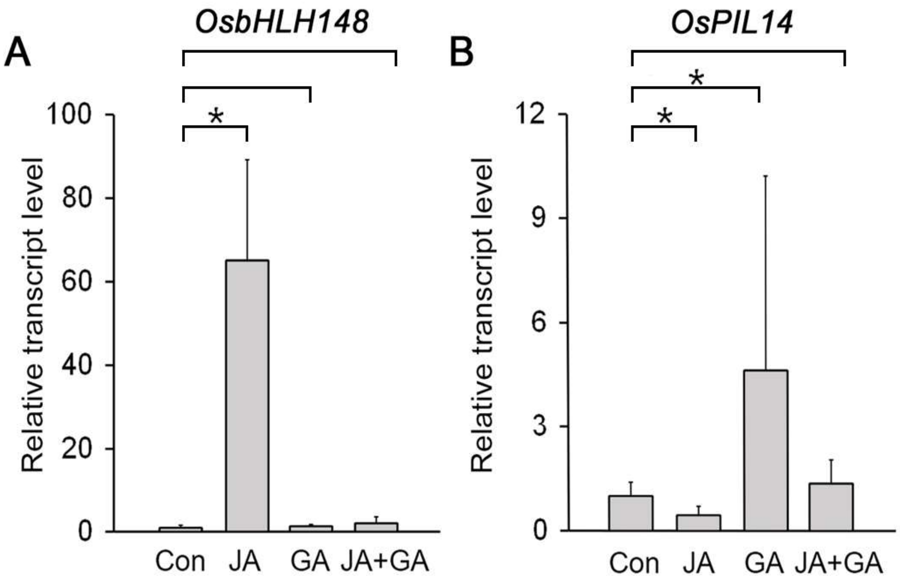


**Supplementary Figure 4.** Expression of *OsbHLH148* and *OsPIL14* in response to JA and GA.
**(A, B)** RT-qPCR analysis showing changes in transcript levels of *OsbHLH148* **(A)** and *OsPIL14* **(B)** in the leaf blade of 8-week-old WT plants (Dongjin) treated with 10 μM MeJA (JA), 10 μM GA3 (GA), or 10 μM MeJA + 10 μM GA3 (JA+GA) for 3 hrs. Control plants (Con) were not treated with JA, GA, or JA+GA. Data represent mean value of three biological replicates, and error bars indicate SD. Asterisks indicate statistically significant differences between the corresponding samples and their respective controls ($p$ value < 0.01, Student's $t$-test). *OsUBI1* was used as an internal control and relative expression levels are shown in fold values.

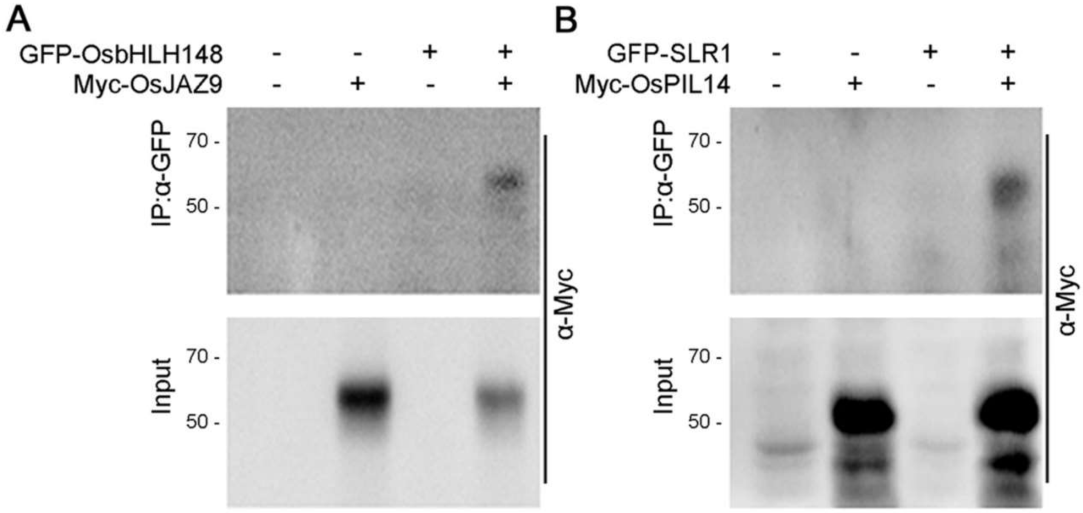


**Supplementary Figure 5.** Interaction between OsJAZ9 and OsbHLH148 or SLR1 and OsPIL14. Co-IP results showing direct interaction between OsJAZ9 and OsbHLH148 **(A)** or SLR1 and OsPIL14 **(B)**. Total proteins were extracted from rice protoplasts co-transformed with 6xMyc-OsJAZ9 and GFP-OsbHLH148 or 6xMyc-OsPIL14 and GFP-SLR1. IP indicates immunoprecipitation. α-GFP was used for the immunoprecipitation of GFP-OsbHLH148 and GFP-SLR1; α-Myc was used for western blotting to detect 6xMyc-OsJAZ9 and 6xMyc-OsPIL14.

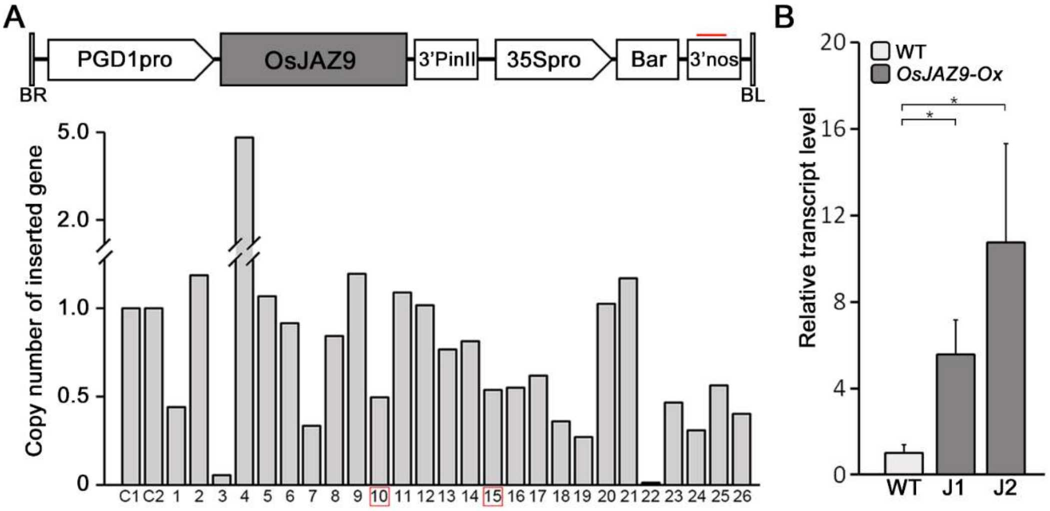


**Supplementary Figure 6.** Analysis of *OsJAZ9*-overexpressing transgenic rice.
**(A)** A schematic of the *PGD1::OsJAZ9* construct for the generation of *OsJAZ9*-overexpressing transgenic rice plants (top) and TaqMan PCR results (bottom) in 26 independent $T_0$ heterozygous transgenic lines. C1 and C2 indicate control plants with a homozygous single-copy transgene including the 3' *nos* terminator. The red line indicates the location of a TaqMan PCR probe used for determining the copy number of the transgene. Red boxes mark lines 10 and 15, which carry a single-copy insertion. Lines 10 and 15 were named line 1 (J1) and line 2 (J2), respectively, and were used in this study. **(B)** Quantitative RT-PCR analysis showing the expression levels of *OsJAZ9* in the leaf blades of 8-week-old *OsJAZ9*-overexpressing transgenic rice (J1 and J2). Data represent mean values of three biological replicates, and error bars indicate SD. *OsTubA2* was used as an internal control and relative expression levels are shown in fold values. Asterisks indicate statistically significant differences between the corresponding samples and their respective controls ($p$ value < 0.01, Student's $t$-test).

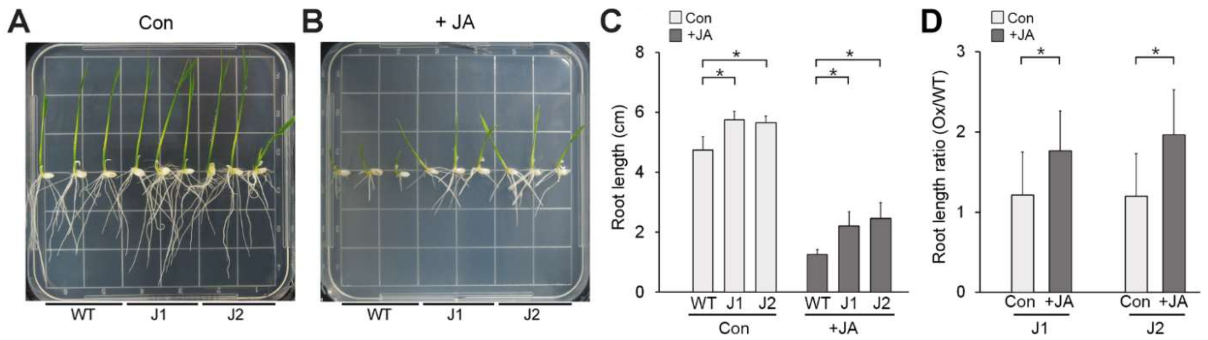


**Supplementary Figure 7.** Root growth of *OsJAZ9*-overexpressing transgenic rice
Images of 7-day-old *OsJAZ9*-overexpressing transgenic rice (*PGD1::OsJAZ9*) grown on 1/2 MS media without JA (Con) **(A)** and with 10 µM MeJA (+JA) **(B)**. **(C, D)** Root growth of these plants ($n$>15) **(C)**, and root length ratio of the transgenic plants to the wild-type plants **(D)**. J1 and J2 indicate two independent lines of *PGD1::OsJAZ9* plants. Data represent mean value, and error bars indicate SD. Asterisks indicate statistically significant differences between the corresponding samples and their respective controls ($p$ value < 0.01, Student's $t$-test).

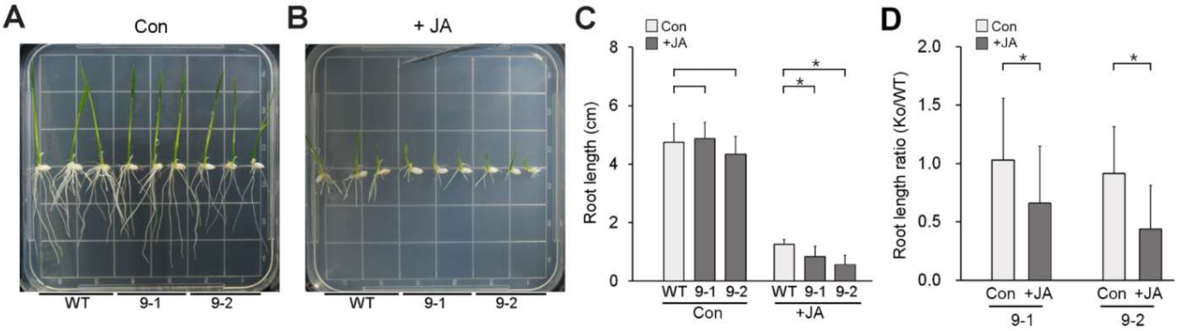


**Supplementary Figure 8.** Root growth of *osjaz9* knockout mutants.
Images of 7-day-old *osjaz9* knockout mutants grown on 1/2 MS media without JA (Con) **(A)** and with 10 µM MeJA (+JA) **(B)**. **(C, D)** Root growth of these plants ($n$>15) **(C)**, and root length ratio of the mutants to the wild-type plants **(D)**. 9-1 and 9-2 indicate two independent *osjaz9* mutant lines. Data represent mean value, and error bars indicate SD. Asterisks indicate statistically significant differences between the corresponding samples and their respective controls ($p$ value < 0.01, Student's $t$-test).

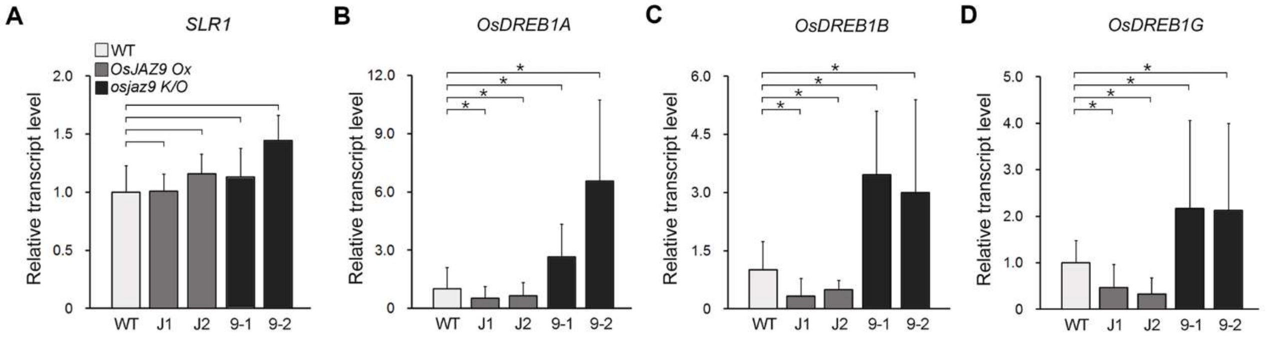


**Supplementary Figure 9.** Expression of *SLR1* and *OsDREB1s* in *OsJAZ9-Ox* and *osjaz9* plants. RT-qPCR analysis showing the expression levels of *SLR1* **(A)**, *OsDREB1A* **(B)**, *OsDREB1B* **(C)**, and *OsDREB1G* **(D)** in the leaf blades of 8-week-old *OsJAZ9*-overexpressing transgenic plants (*PGD1::OsJAZ9*) and the *osjaz9* mutants. J1 and J2 indicate two independent lines of *PGD1::OsJAZ9*, and 9-1 and 9-2 indicate two independent *osjaz9* mutants. Data represent mean value of three biological replicates, and error bars indicate SD. Asterisks indicate statistically significant differences between the corresponding samples and their respective controls (p value < 0.01, Student's t-test). *OsTubA2* was used as an internal control and relative expression levels are shown in fold values.

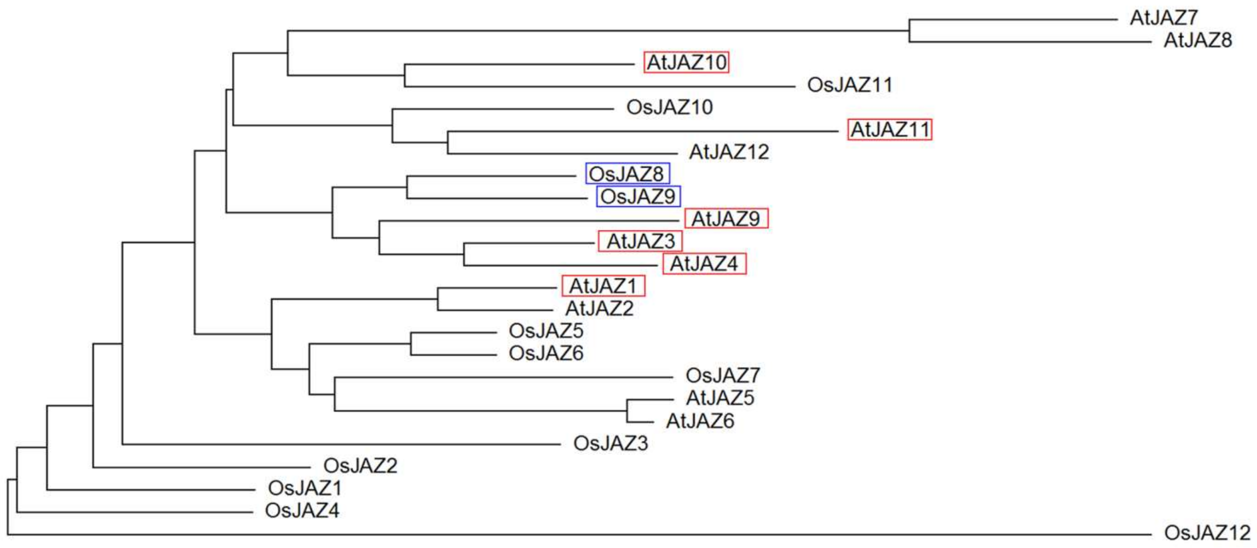


**Supplementary Figure 10.** Relationship between Arabidopsis JAZs and rice JAZs.
A phylogenetic tree showing the relationship between Arabidopsis JAZs and rice JAZs. Full-length amino acid sequences of OsJAZs and AtJAZs were used for the tree. OsJAZ8 and OsJAZ9, which interact with SLRs, are closely related to the Arabidopsis JAZs that interact with RGA. Blue and red boxes indicate the OsJAZs and AtJAZs that interact with DELLAs.

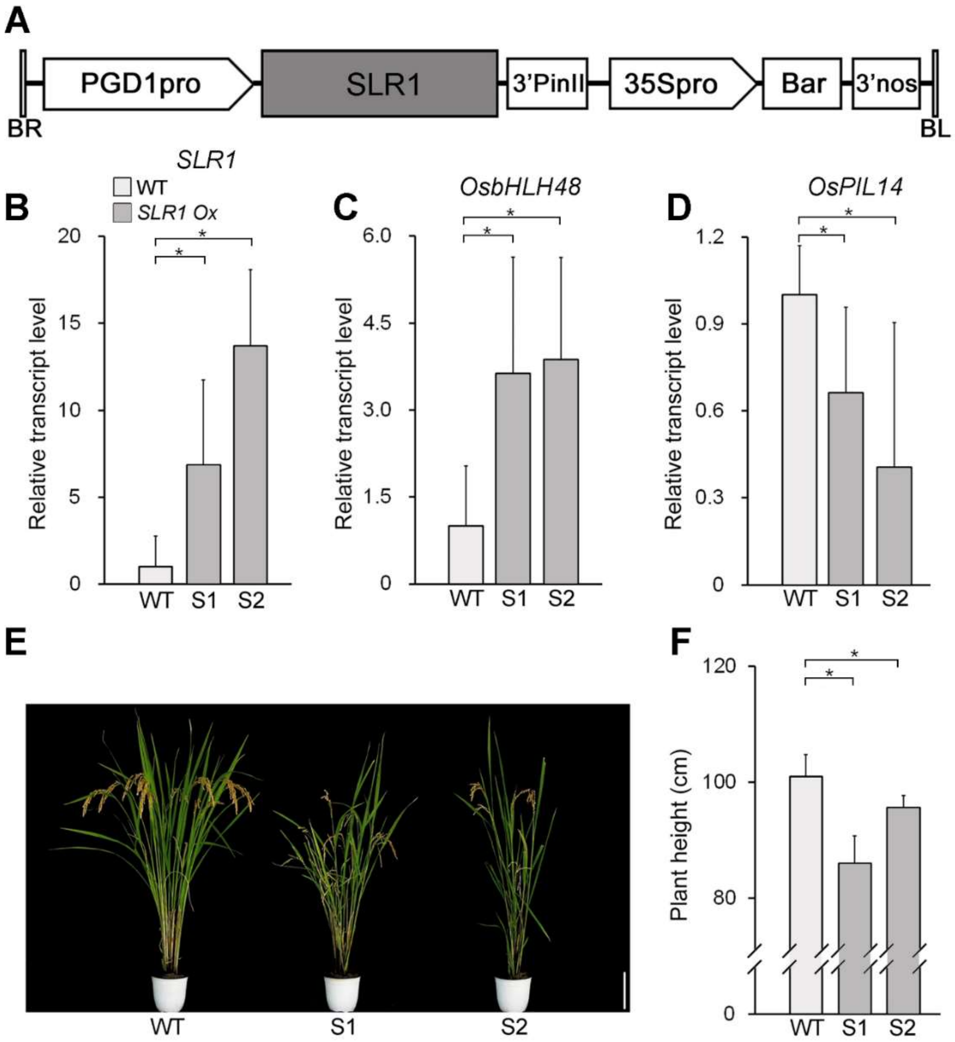


**Supplementary Figure 11.** Analysis of *SLR1*-overexpressing transgenic rice.
**(A)** A schematic of the *PGD1::SLR1* construct for the *SLR1*-overexpressing transgenic rice plants. **(B-D)** RT-qPCR analysis showing the expression levels of *SLR1* **(B)**, *OsbHLH148* **(C)** and *OsPIL14* **(D)** in the leaf blades of 8-week-old *SLR1*-overexpressing transgenic rice (S1 and S2). Data represent mean value of three biological replicates, and error bars indicate SD. *OsTubA2* was used as an internal control and relative expression levels are shown in fold values. **(E, F)** Growth of 20-week-old *PGD1::SLR1* plants **(C)**, and quantification of plant height (*n* >30) **(D)**. Error bars indicate SD. Asterisks indicate statistically significant differences between the corresponding samples and their respective controls (*p* value < 0.01, Student's *t*-test).

**Supplementary Table 1.** Primers used in this study

| Gene | Primer Sequence | |
|---|---|---|
| | **Forward (5' to 3')** | **Reverse (5' to 3')** |
| **For overexpressing transgenic plants** | | |
| OsJAZ9 | GGATCCATGGAGAGGGATTTT | CCCGGGTCATATCTGTAACTTTG |
| **For yeast two-hybrid** | | |
| RGA1 | CATATGATGAAGAGAGATCATCACCAATTCCAA | GAATTCTCAGTACGCCGCCGTCGA |
| SLR1 | CATATGATGAAGCGCGAGTACCAAG | GAATTCTCACGCCGCGGCGAC |
| SLRL1 | CATATGATGGCCATGGATACCTTCCC | GAATTCTTACAAACACACGCTGCTACCAT |
| SLRL2 | CATATGATGGCTCAGTTCGGCGGC | GAATTCTCACTGCATGATTTGGTTGAGAGC |
| OsJAZ1 | CATATGATGGCCGCCGCCGGC | GGATCCTCAGAGCCCGAGCCATGTCG |
| OsJAZ2 | CATATGATGGCCGGTAGTAGCGAG | GGATCCTCACAGGCTGAGAGTGGG |
| OsJAZ3 | CATATGATGGCGTCGACGGA | GAATTCTCAGCGCGAGTGCA |
| OsJAZ4 | CATATGATGGCGATGGAGGGGAG | GGATCCTCACAGCGCGATGGTGA |
| OsJAZ5 | CATATGATGGCTTCCGCGAAATC | GGATCCTCATTGGCTCGATTCCTG |
| OsJAZ6 | CATATGATGGCGGCTTCCGCGAG | GGATCCTCATTGGCCGCGTTCTATGG |
| OsJAZ7 | CATATGATGGCCGGCCGTGC | GGATCCTCATATCTCCTGCTTTA |
| OsJAZ8 | CATATGATGGAGAGGGACTTC | GGATCCTCAGATTTGTAGCTTTG |
| OsJAZ9 | CATATGATGGAGAGGGATTTT | GGATCCTCATATCTGTAACTTTG |
| OsJAZ10 | CATATGATGTCGAGCCCTCCACC | GGATCCTTACTGGGCCTTGCCCT |
| OsJAZ11 | CATATGATGTCGACGAGGGC | GAATTCCTAGGACGCCGTGTG |
| OsJAZ12 | CATATGATGCCGCCGCCG | GAATTCTCACCAGCAATGAGACATC |
| SLR1D | CATATGATGAAGCGCGAGTACCAAG | GAATTCGTCAACCACCACAACCGG |
| SLR1G | CATATGCCGGTTGTGGTGGTTGA | GAATTCTCACGCCGCGGCGAC |
| JAZ9N | CATATGATGGAGAGGGATTTT | GAATTCATTTTGAAGGTCCCTTGG |
| JAZ9Z | CATATGCCAAGGGACCTTCAAAAT | GAATTCTTGCGACACGATGGTGAT |
| JAZ9J | CATATGATCACCATCGTGTCGCAA | GGATCCTCATATCTGTAACTTTG |
| JAZ9NZ | CATATGATGGAGAGGGATTTT | GAATTCTTGCGACACGATGGTGAT |

| | | |
|---|---|---|
| **For Co-IP** | | |
| OsJAZ9 | TCCGGAAGCCCGAGGATGGAGAGG GATTTTCTTG | AATGTTTGAACGATCTCATATCTGT AACTTTGTGC |
| SLR1 | CTGTACAAGGCGGCCATGAAGCGC GAGTACCAAG | AATGTTTGAACGATCTCACGCCGC GGCGAC |
| **For BiFC** | | |
| OsJAZ9 | GAATTCAATGGAGAGGGATTTT | CCCGGGTCATATCTGTAACTT |
| SLR1 | GAATTCAATGAAGCGCGAGTA | CCCGGGTCACGCCGCGGCGAC |
| **For qRT-PCR** | | |
| OsJAZ9 | CATTCCGGTGGAGAAGGCTC | GGGCACTGTAAGTTTCGCAG |
| SLR1 | CTGGAGAAGGTCCTGGGCAC | TGAACCGGTCGAGGAATGAG |
| OsPIL14 | ATGATGTTCCCCGGTACCCA | GGCATCTGGTTTGGCAGAGA |
| OsbHLH148 | ATGAACGTCGAGACCACGATTGC | ACATTTTGCACATGAGATGTCTGTG TCC |
| OsDREB1A | GACGTCCTGAGTGACATGGG | AGTAGCTCCAGAGTGGGACG |
| OsDREB1B | CGCACTGAAAAGTGTGGACAA | GGAGGGAGAAATCTGGCACA |
| OsDREB1G | CTTGGGGATGCAGGGCTATC | TCAAACGCGCAGGATCAGTA |
| OsTubA2 | TCCGAGTTCGACGATGGTGACG | AGCCCACACGGACAGATCATAGGA TA |
